# Improvement in resolution of fiber-laser photoacoustic tomography based on a virtual-point concept

**DOI:** 10.1186/s42492-021-00070-4

**Published:** 2021-02-23

**Authors:** Xue Bai, Xu Li, Jun Ma, Bai-Ou Guan

**Affiliations:** Guangdong Provincial Key Laboratory of Optical Fiber Sensing and Communications, Institute of Photonics Technology, Jinan University, Guangzhou, 511443 China

**Keywords:** Photoacoustic tomography, Fiber laser, Virtual point, Synthetic aperture focusing

## Abstract

In this study, a virtual-point concept was introduced into fiber-laser photoacoustic tomography to improve the elevational image resolution. The flexible fiber laser was bent into an arc shape to conform to the ultrasound wavefront, which formed an ultrasound focus at the center of the arc. The synthetic aperture focusing technique was utilized to reconstruct the images; as a result, the elevational resolution particularly within the out-of-focus region was considerably improved compared to the resolution of an image retrieved by multiplexing the PA time-resolved signals with sound velocity. The all-optical fiber-laser photoacoustic tomography system with a high spatial resolution has potential for various applications, including biomedical research and preclinical/clinical diagnosis.

## Introduction

Photoacoustic tomography (PAT) is a hybrid, noninvasive biomedical imaging technology that combines the high contrast of optical imaging and deep penetration of ultrasonography [1–6]. Photoacoustic (PA) signals generated from an absorber irradiated using a short-pulse laser are detected to acquire the structural and functional information of the biological tissues. The absorber can be endogenous in biological tissues, for example, hemoglobin, melanin, and lipids, or an exogenous contrast agent such as dyes and nanomaterials [7–9]. Most PAT systems detect PA signals using piezoelectric (PZT) ultrasound transducers that are sensitive to electromagnetic interference (EMI) and have limited acceptance angles because of their large aperture sizes [10]. A small acceptance angle inevitably causes artifacts and degrades the image quality. Although small-sized transducers theoretically have larger acceptance angles, their sensitivity is low because of the smaller active element and higher thermal noise level. To address this issue, the focus of the ultrasound transducer was treated as a virtual point detector to increase the acceptance angle. This concept was verified using a flat PZT transducer with an element surface attached using a negative lens [11]. To avoid image artifacts induced by the reverberations of ultrasonic waves between the lens and the transducer surface, Nie et al. directly applied a custom-built PZT transducer with a convex surface geometric shape to image a monkey brain [12]. Lin et al. recently demonstrated the use of a specially designed dual-foci transducer with two foci along two perpendicular directions. One focus acts as the virtual point to increase the tangential resolution and the other enhances the sensitivity in the elevational direction [13].

Compared to rigid PZT materials, optical fibers are highly flexible and immune to EMI [14–16], and fiber-based ultrasound transducers have the advantages of a high sensitivity per unit area, wide frequency bandwidth, and optical transparency [17–19]. For example, a fiber-optic Fabry–Pérot (FP) etalon, formed using a polymer film spacer that was sandwiched between a pair of highly reflective mirrors at the fiber tip facet, was employed as an ultrasound detector for wide-view imaging of mouse ears [17]. The FP etalon can also be formed through the in-line inscription of two fiber Bragg gratings (FBGs), wherein the space between the gratings can be used as the ultrasound sensing element [18]. To increase the sensitivity, a pi-phase-shifted FBG featuring a sharp notch in the reflection spectrum used a frequency comb source for imaging zebrafish larvae [19]. All of the optical fiber transducers described above are flat or line-shaped [18–20]. The sensitivity decreases rapidly with an increase in the distance between the target and the transducer surface. We previously developed a curved fiber-laser ultrasound transducer for the circular-scanning photoacoustic computed tomography (PACT) imaging of a zebrafish and a mouse brain. The fiber was bent into an arc shape to realize ultrasound focusing with a centimeter-scale focal length for deep-tissue imaging [21]. The focal length of the fiber laser transducer was varied by tuning the fiber bending curvature with a lab-designed holder for the multi-depth linear-scanning PACT imaging of a rat abdomen [22]. However, the elevational resolution rapidly decays within the out-of-focus region for the focused fiber-laser transducer, which degrades the sectioning capability for high-resolution cross-sectional imaging or slice-stacking 3D imaging.

Herein, we introduce the virtual-point concept into a fiber-laser linear-scanning PAT to improve the elevational resolution of the image. The flexible fiber laser was bent into an arc shape to conform to the ultrasound wavefront and form an ultrasound focus at the center of the arc. The focus was treated as a virtual point detector to reconstruct the image from the acquired PA time-resolved signals at each scanning point. Simulated and experimental results demonstrated that the image reconstructed from the virtual-point concept-based synthetic aperture focusing technique (SAFT) significantly improved the elevational resolution, particularly within the out-of-focus region.

## Method

### Fiber-laser ultrasound transducer-based PAT system

**Fig. 1:**
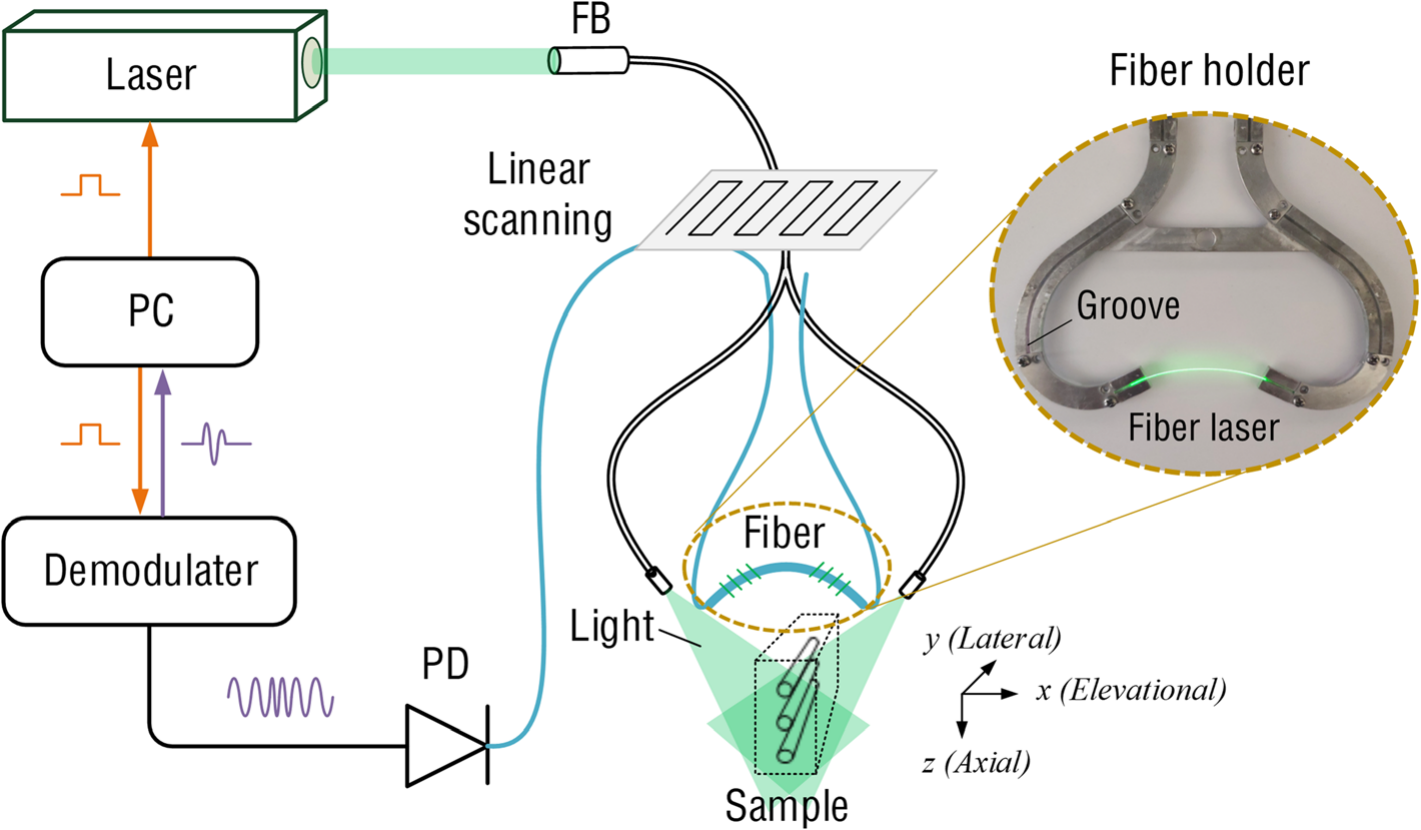
Schematic of a linear-scanning PAT system with a focused fiber-laser ultrasound transducer: FB, fiber bundle; PC, personal computer; PD, photodetector

Figure 1 shows the configuration of the PAT system using a focused fiber-laser ultrasound transducer. The fiber was bent using a custom-made fiber aluminum holder, as shown in the inset of Fig. 1. The arc-shaped fiber laser conforms to the ultrasound wavefront and forms a focus at the arc center. A 532-nm Nd:YAG laser (Dawa 100, Beamtech) with a pulse width of 6.5 ns and repetition rate of 10 Hz was used as the excitation source. The pulsed light output after the collimation was coupled into a customized 5-mm-diameter Y-shaped fiber bundle consisting of 480 fibers with a core diameter of 190 μm. The bundle exhibited a light coupling efficiency of approximately 60%. The average optical fluence on the target surface is ∼3 mJ/cm^2^, which is within the maximum permissible exposure (MPE) of 20 mJ/cm^2^ recommended by the American National Standards Institute (ANSI). The imaging sample was immersed in water and irradiated by a laser beam from the top, and the focused fiber laser ultrasound transducer was linearly scanned along the sample. Synchronized clock signals from a control board were used to trigger the laser and data acquisition (DAQ) system. The PA signals were detected using a fiber-laser transducer, and the output optical signals were received by a high-speed photodetector (DSC50S-39-FC, Discovery Semiconductor, Inc.). The converted electrical signals were then demodulated using a vector signal analyzer (Pxie-5646R, NI) for subsequent image processing.

### Ultrasound detection

**Fig. 2:**
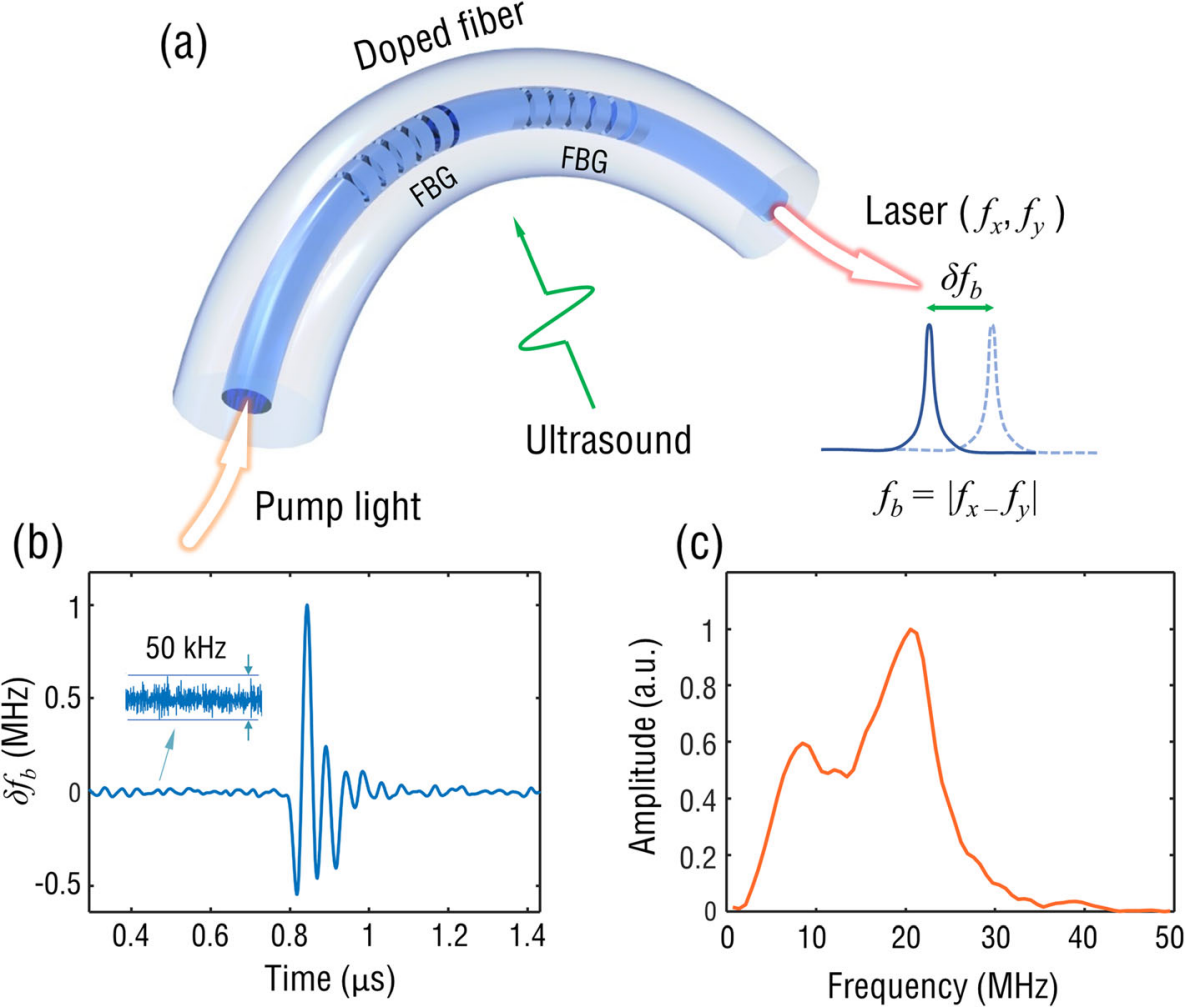
**a** Work principle of fiber-laser ultrasound transducer. **b** Temporal response and **c** frequency response to an ultrasound pulse

A schematic of the fiber laser ultrasound transducer is shown in Fig. 2a. Two Bragg gratings were inscribed into an Er/Yb-doped fiber using 193-nm UV light to form an inline Fabry-Perot cavity. When 980 nm pump light enters the fiber, two orthogonal polarization modes (*f*_*x*_ and *f*_*y*_) at 1530 nm generated a beat signal (*f*_*b*_ = | *f*_*x*_ – *f*_*y*_ |) as a result of the intrinsic birefringence in the fiber. As ultrasound waves impinge onto the fiber, the stress-induced birefringence results in a frequency shift of the beat signal, which can be read out through I/Q phase demodulation [22]. Figure 2b shows the PA signal detected using a focused fiber-laser ultrasound transducer. Based on the previously measured ultrasound sensitivity of 2.25 MHz/kPa and a noise floor of approximately 50 kHz [22], as estimated from Fig. 2b, the noise-equivalent pressure (NEP) can be calculated as approximately 25 Pa for a measurement bandwidth of 50 MHz. Figure 2c shows the frequency response after the Fourier transform of the time-domain PA signal shown in Fig. 2b. The frequency response of the transducer had a peak at approximately 22 MHz and a bandwidth of approximately 20 MHz.

### Synthetic aperture focusing technique (SAFT)

**Fig. 3:**
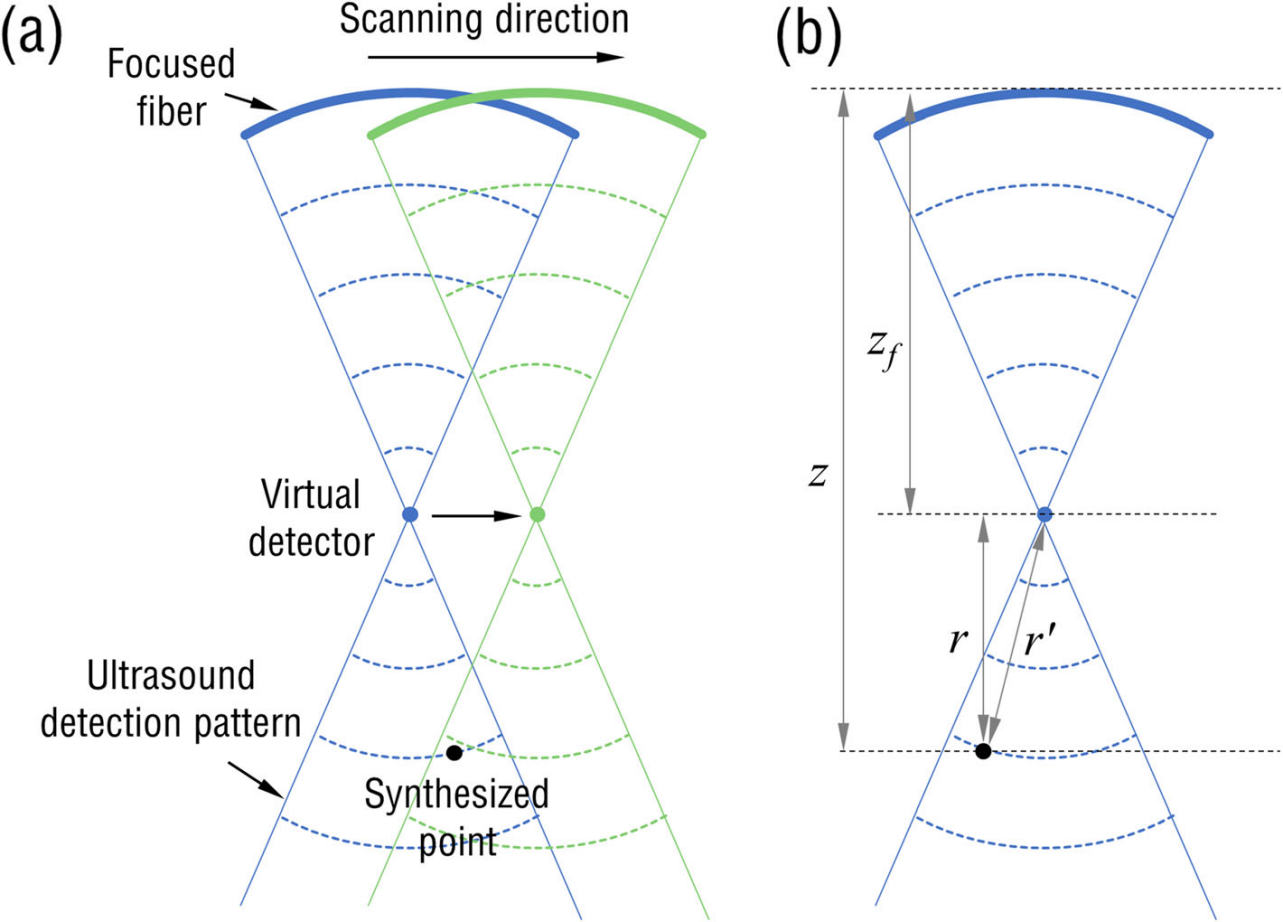
**a** Schematic of a virtual point detector for SAFT. **b** Focusing geometry for the SAFT to calculate the time delay ∆*t*_*i*_

The virtual point concept was combined with the SAFT to improve the degraded spatial resolution in the out-of-focus region for focused PZT transducers [23, 24]. Here, the fiber-laser transducer can be regarded as a lens-free focused transducer with a focal point coinciding with the arc center of the curved fiber laser. The principle of the virtual-point-based SAFT is illustrated in Fig. 3a. For a focused transducer, only a part of the spherical photoacoustic waves emitted from the source within a limited angle can be detected by the transducer due to the limited acceptance angle. Therefore, synthetic-aperture focusing can be applied to the source located at the overlap region of the detection pattern of the transducer at adjacent scanning positions during linear-scanning motion. The virtual-point-based SAFT is conducted by applying appropriate time delays that correspond to the received PA time-resolved signals at each scanning position (or A-lines) and then calculating the sum of the delayed A-lines. The procedures can be described through the following expression [25]:

1 $$ {RF}_{SAFT}(t)=\sum \limits_{i=0}^{N-1} RF\left(i,t-\Delta  {t}_i\right), $$

where *RF*(*i, t*) is the detected PA signal at the *i-*th position, and *N* denotes the total number of adjacent scan lines included in the SAFT summation, which is determined by the detection pattern of the transducer. ∆*t*_*i*_ denotes the time delay applied to the signal of scan line *i*, which can be computed using

2 $$ \Delta  {t}_i=\mathit{\operatorname{sign}}\left(z-{z}_f\right)\bullet \frac{r^{\prime }-r}{c}, $$

where *c* is the velocity of sound; *z* and *z*_*f*_ are the depths of the synthesized and virtual points, respectively. *r’* and *r* are the distance from the synthesized point to the virtual point and the depth difference between the two points, respectively, as shown in Fig. 3b.

## Results

### Elevational resolution improvement

**Fig. 4:**
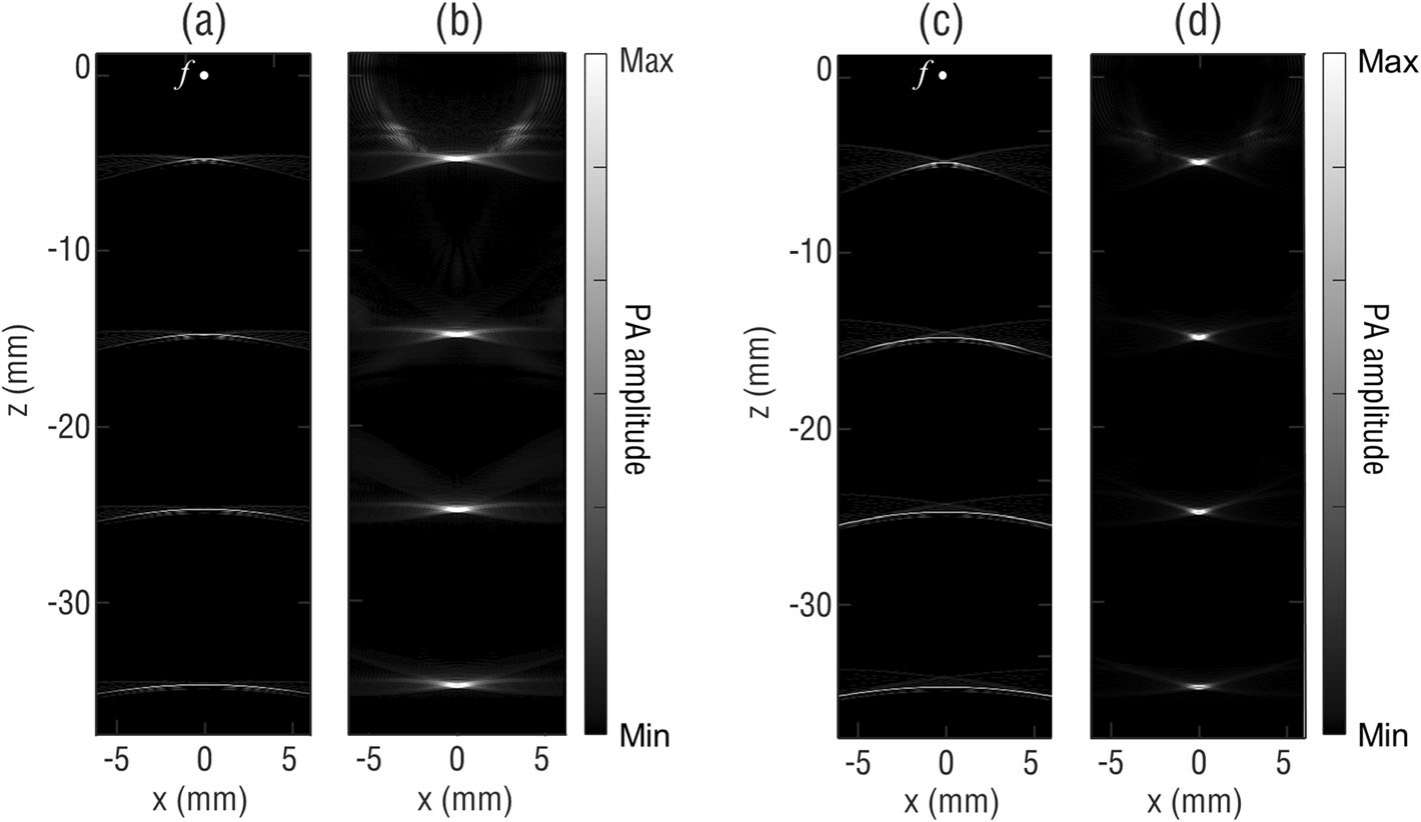
Simulated B-scan and SAFT images detected using a focused fiber with an NA of 0.18 (**a, b**) and 0.36 (**c, d**)

To verify the improvement in elevational resolution by the virtual-point SAFT, the k-wave MATLAB® toolbox was used to simulate the spatial response of the focused fiber laser to ultrasound. The fiber laser in the simulation had a curvature radius of 27 mm and a cavity length of approximately 10 mm, which was chosen in accord with the parameters of the fiber laser used during the experiment. The numerical aperture (NA) of the fiber laser can then be calculated as approximately 0.18. The frequency bandwidth of the fiber laser was set according to the measured frequency response, as shown in Fig. 2c. To analyze the spatial ultrasound response of the fiber laser, four spherical sources with the same radius of 80 μm were positioned along the *z-*axis at every 10 mm, and the first source was placed 5 mm below the focus of the fiber laser. The fiber laser was linearly scanned with a step size of 200 μm over a distance of 12 mm. Figure 4a shows the B-scan image obtained by directly converting the obtained A-line signals to depth-resolved signals at each scanning point by multiplying the ultrasound velocity. The image of the absorbers indicates that the *x*-axis (or elevational) resolution deteriorates rapidly with increasing distance from the focus. In contrast, the imaging resolution of the virtual-point SAFT remains nearly the same at different depths along the z-axis, as shown in Fig. 4b. Figure 4c and 4d show the simulated results for a fiber laser with a cavity length of 20 mm, which suggests that a larger NA of 0.36 can provide a better elevational resolution.

**Fig. 5:**
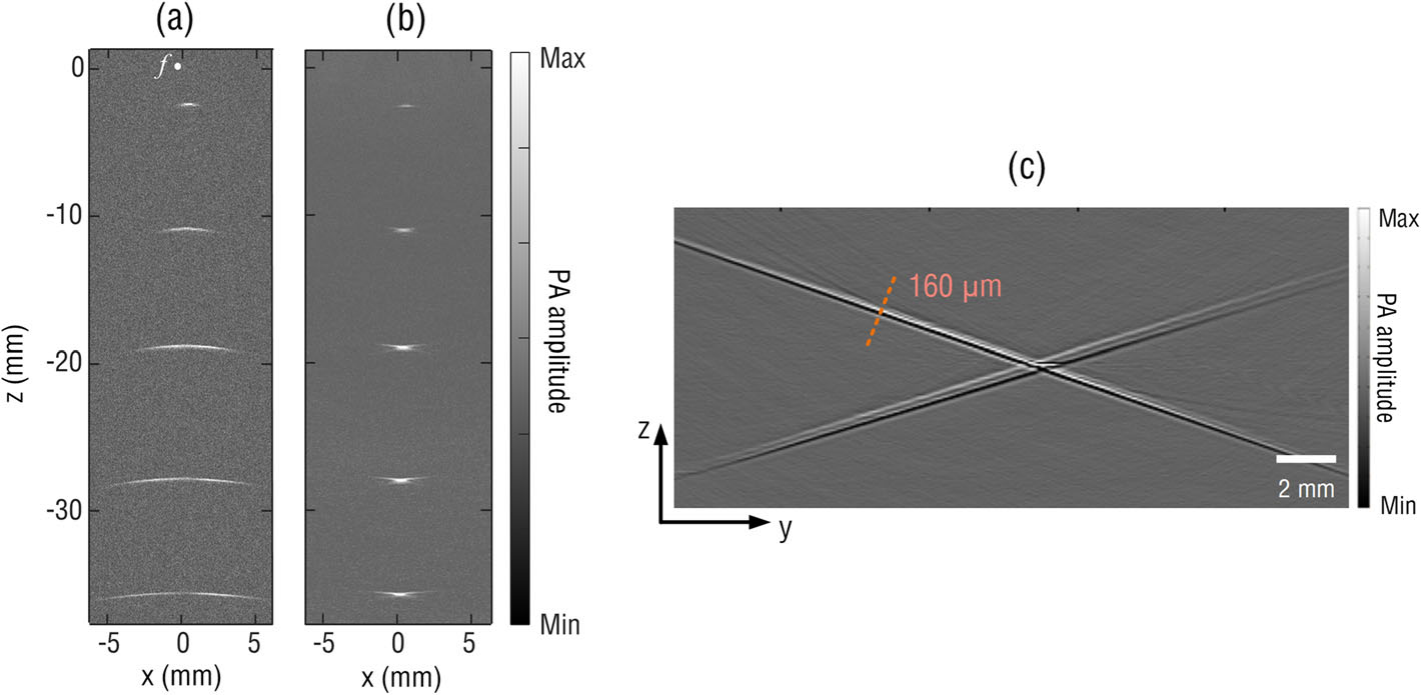
Measured (**a**) B-scan and SAFT (**b**) images, and image of a hair cross on the *y-z* plane (**c**)

To validate the simulation results, a fiber laser with geometrical parameters similar to those used in the simulation was employed for the imaging of a phantom that was specifically designed to simulate the point ultrasound source. The phantom consisted of five human hairs with an average diameter of approximately 80 μm. The hairs were aligned in parallel in a block of agar with a spacing of approximately 10 mm along the *z-*axis (see Fig. 1). The longitudinal direction of the hairs was perpendicular to the B-scan imaging or the *x-z* plane. PA signals were acquired at a sampling rate of 100 MS/s, without additional averaging or interpolation. During the imaging process, the fiber laser was linearly scanned along the *x-*axis with a step size of 10 μm. Figure 5a and 5b show the B-scan images before and after using the SAFT, respectively. Similar to the simulated results in Fig. 4, the SAFT can efficiently improve the elevational resolution, particularly at depths within the out-of-focus region, which agrees well with the study using a focused PZT transducer [24].

To quantitatively compare the elevational resolution before and after the SAFT, we measured the full width at half maximum (FWHM) of the hair profiles along the *x*-axis, as summarized in Table 1. The elevational resolution for imaging using the SAFT shows no dependence on the imaging depth, varying only slightly from 0.64 to 0.7 mm. In contrast, the elevational resolution of the hair in the B-scan image varies from 0.43 to 8 mm and degrades significantly with the imaging depth. In addition to the elevational resolution, we chose a hair cross (approximately 80 μm diameter) as the sample to evaluate the in-plane (*y*-*z*) resolution of the system, for which the fiber laser was linearly scanned along the *y-*axis with a step size of 10 μm. Because the fiber laser has a compact size with a diameter of 125 μm, it has a large acceptance angle of approximately 60°. In combination with a broad bandwidth of approximately 20 MHz, the fiber-laser PAT system can provide a high in-plane resolution of 160 μm, as shown in Fig. 5c. This high in-plane resolution, together with the SAFT-improved elevational resolution of the fiber laser system, is promising for future high-quality 3D imaging using 2D mechanical scanning [5, 25].

**Table 1:** Comparison of the elevational resolution before and after SAFT

Systems and improved resolution	Depth from the focus (mm)				
	2.5	11.5	19.1	28.6	37.1
Original system (elevational, mm)	0.43	1.75	3.6	6.4	8
SAFT system (elevational, mm)	0.64	0.69	0.7	0.68	0.7

Unlike point-like ultrasound detectors that generally have sensitivities less than satisfactory, the virtual-point-based SAFT is an efficient method to improve the imaging resolution for focused ultrasound transducers with high sensitivity. As shown in Fig. 4c and d, a larger NA can provide a better resolution when the same SAFT procedure is applied. One approach to increase the NA of the fiber laser is to increase its cavity length. However, this causes mode competition and instability of the laser output. Hence, we attempted to fabricate long-cavity single-longitudinal-mode lasers by inserting in-line fiber-optic filters, such as phase-shift fiber Bragg gratings, into the laser cavities to suppress the mode competition. As a proof-of-concept demonstration of the virtual-point concept for a focused fiber laser, a fiber laser with a relatively short cavity length of approximately10 mm was used in this study. Another approach to enlarge the NA is to bend the fiber into a smaller curvature radius. Currently, the minimum curvature radius of the fiber laser without breakage is approximately20 mm, which can be further reduced if the coating-stripped region of the fiber laser is recoated.

## Conclusions

In summary, we demonstrated a fiber-laser virtual-point detector by bending the fiber into arcs. The curved fiber laser with its focus in the arc center, combined with the synthetic aperture focusing technique, significantly improved the elevational resolution at a depth deviating from the focus. By using a fiber laser with a long cavity, a high numerical aperture or tight focus can be obtained to further increase the spatial resolution of the imaging system in the future. Compared with piezoelectric transducers, the reported detector is sensitive, immune to electromagnetism, weight-light, and flexible, making it attractive to develop high-resolution PACT systems for biomedical studies and clinical diagnosis of breast and skin cancers.

## Data Availability

The datasets generated and/or analyzed during the current study are not publicly available for privacy reasons but are available from the corresponding author upon reasonable request.

## Abbreviations

PAT: Photoacoustic tomography
PA: Photoacoustic
PZT: Piezoelectric
EMI: Electromagnetic interference
VPD: Virtual-point detector
3D: three-dimensional
FP: Fabry–Pérot
FBG: Fiber Bragg gratings
SAFT: Synthetic aperture focusing technique
ANSI: American National Standards Institute
MPE: Maximum permissible exposure
DAQ: Data acquisition
NA: Numerical aperture
NEP: Noise-equivalent pressure
FWHM: Full width at half maximum
